# Supplementary material for: Function of Host Protein Staufen1 in Rabies Virus Replication
Source: Viruses. 2021 Jul 22;13(8):1426. doi: 10.3390/v13081426 (PMC8402631; doi:10.3390/v13081426)
Supplement: Supplementary file 1 [file viruses-13-01426-s001.zip › viruses-1270057-supplementary.pdf]

Figure S1. The effect of STAU1 overexpression on the replication on RABV.

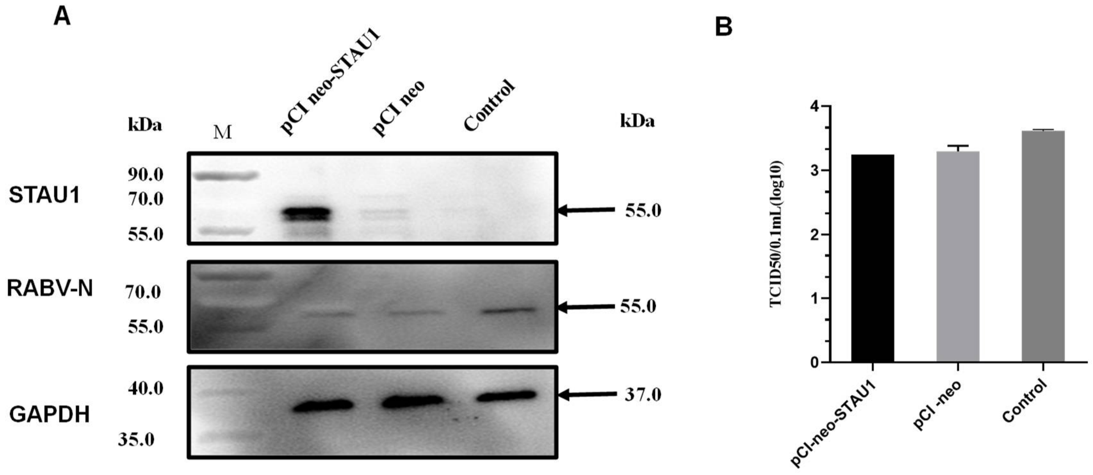

FigureS1. (**A**) Western blot confirmation of cells that overexpress STAU1 and were infected with RABV. (**B**) $TCID_{50}$ detection of RABV after cells upregulate STAU1.

Figure S2. Subcellular distribution of Negri body-like structures formed in RABV-infected LV-shRNA-iSTAU1/LV-shRNA-iC cells.

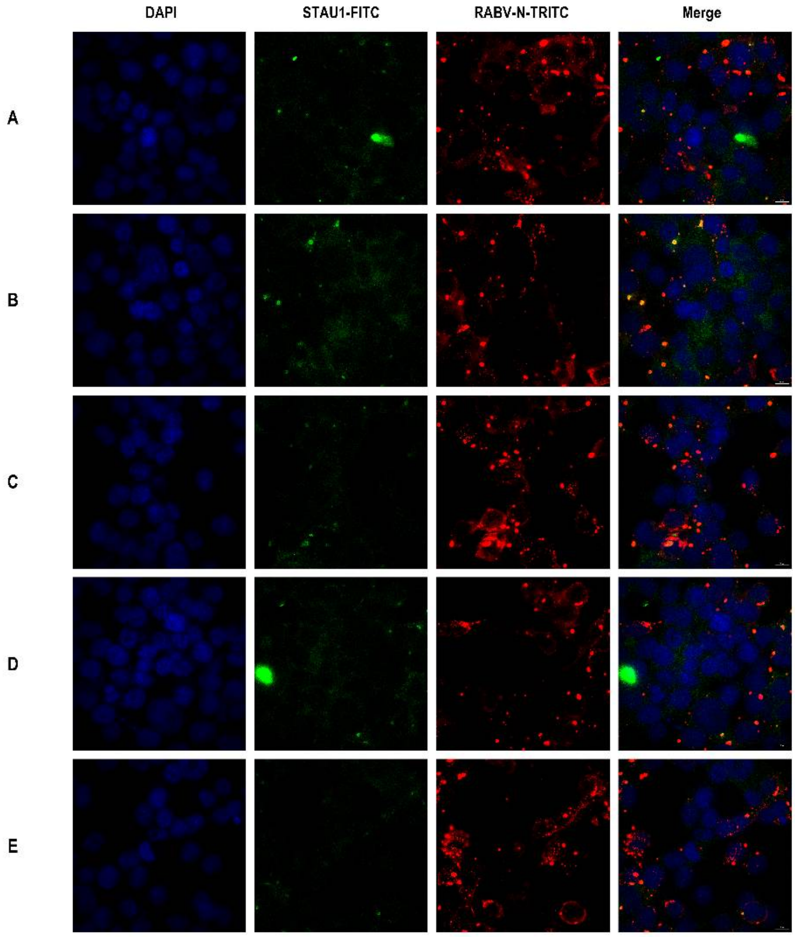

Figure S2A. Subcellular distribution of STAU1 in RABV-infected LV-shRNA-iSTAU1, Bars=10 μm.

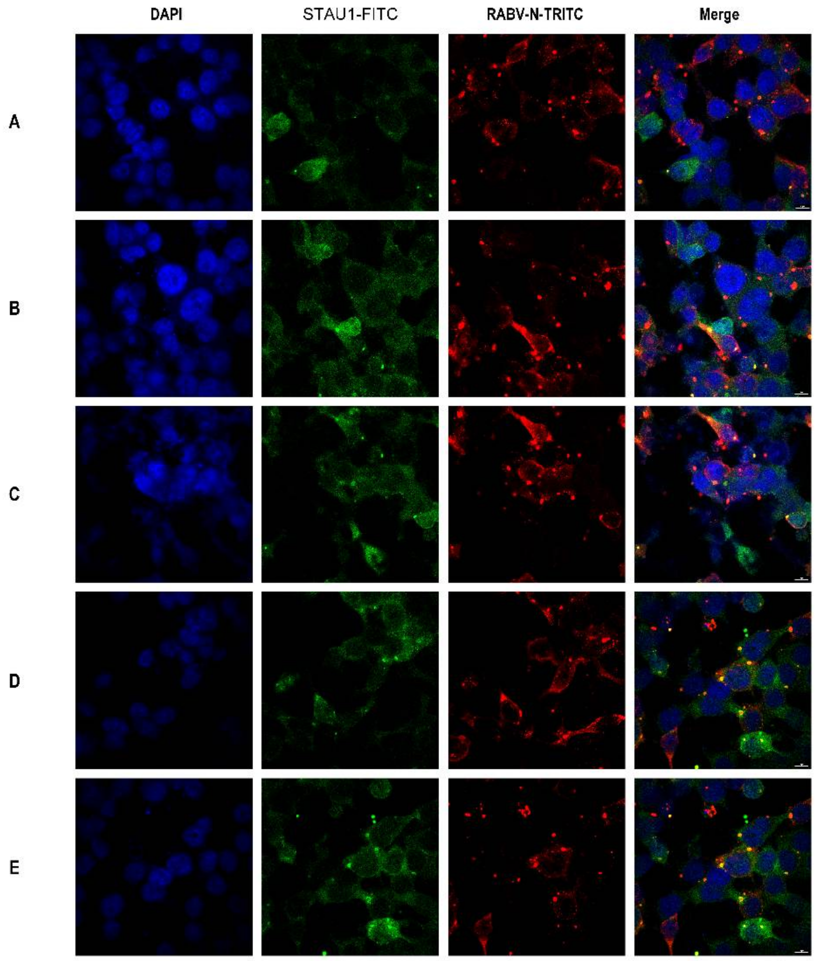

Figure S2B. Subcellular distribution of STAU1 in RABV-infected LV-shRNA-iC, Bars=10μmm.
